# Supplementary figures and images for: Strong spurious transcription likely contributes to DNA insert bias in typical metagenomic clone libraries
Source: Microbiome. 2015 May 20;3:22. doi: 10.1186/s40168-015-0086-5 (PMC4459075; doi:10.1186/s40168-015-0086-5)

crude extract F

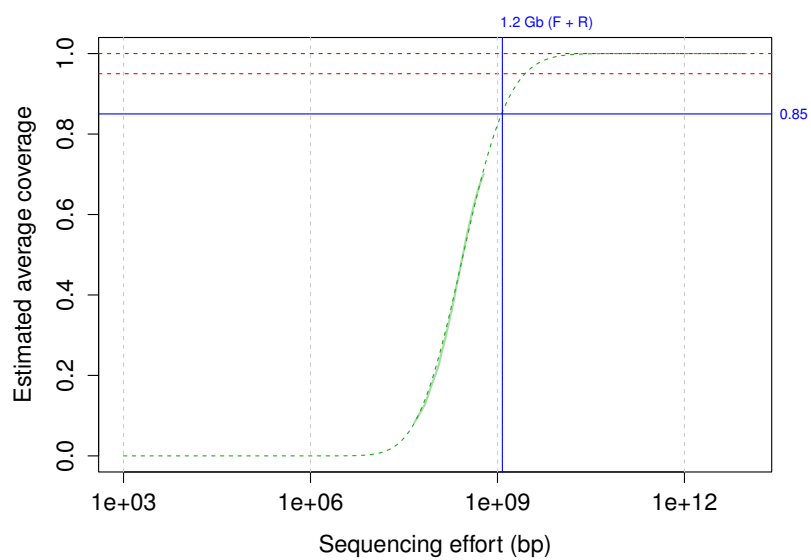

size-selected F

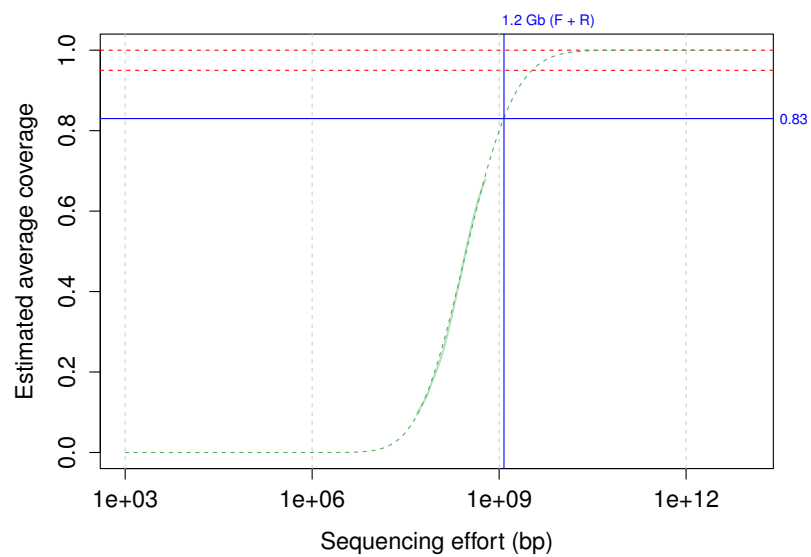

cosmid library F

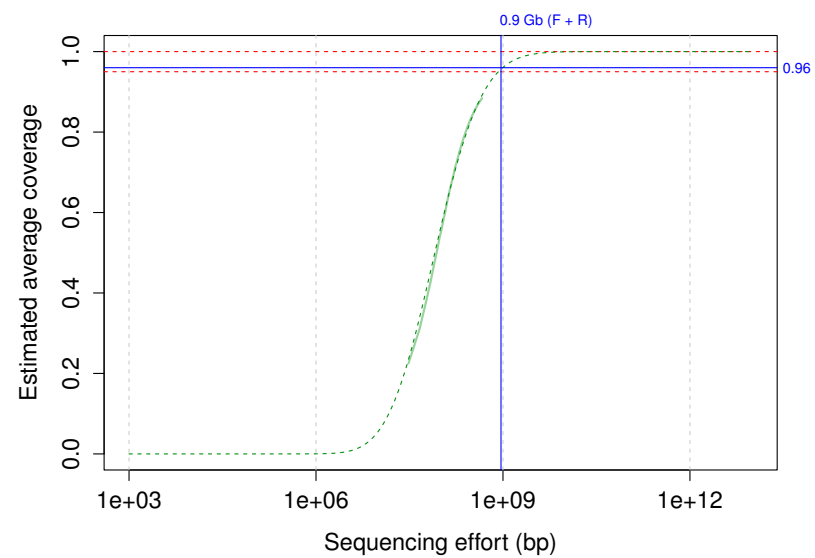

Supplement: Additional file 1: Figure S1. — Estimate of sample sequencing coverage using Nonpareil. [file 40168_2015_86_MOESM1_ESM.pdf]
