# Supplementary material for: Strong spurious transcription likely contributes to DNA insert bias in typical metagenomic clone libraries
Source: Microbiome. 2015 May 20;3:22. doi: 10.1186/s40168-015-0086-5 (PMC4459075; doi:10.1186/s40168-015-0086-5)

crude extract F

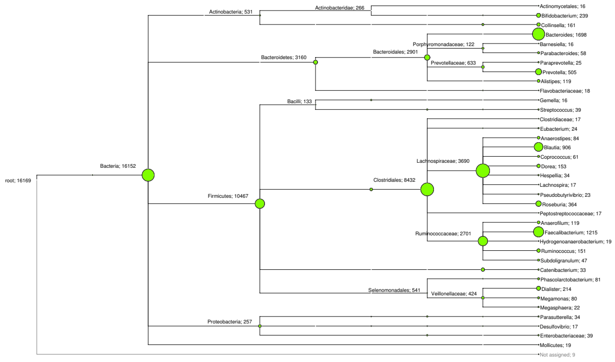

crude extract R

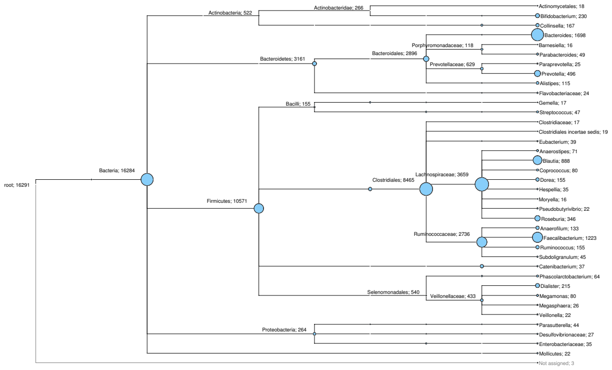

size-selected F

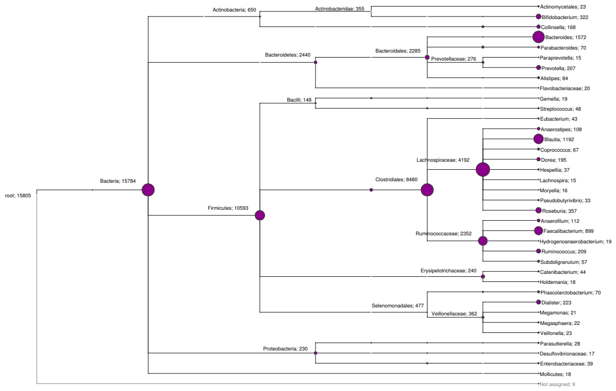

size-selected R

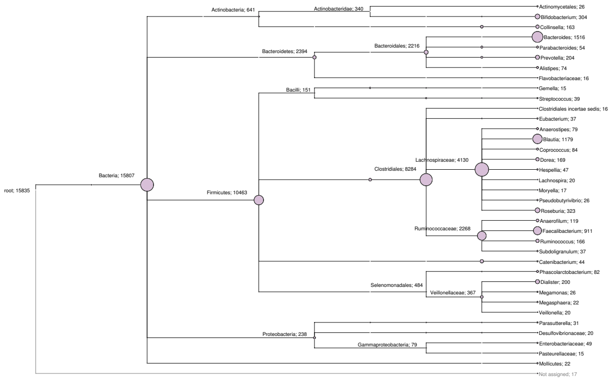

cosmid library F

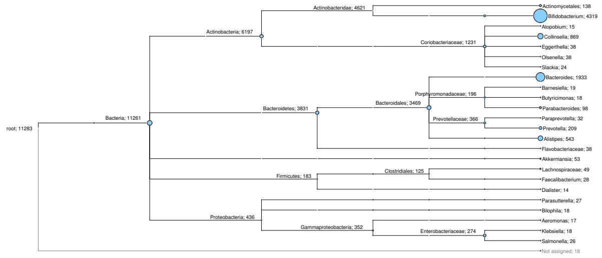

cosmid library R

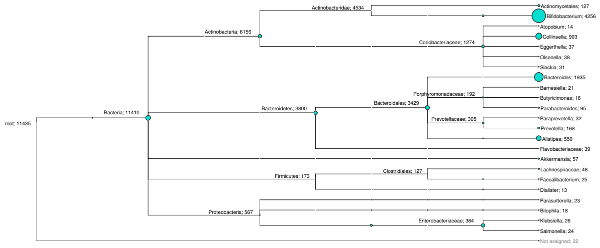

Supplement: Additional file 3: Figure S2. — 16S rRNA analysis results using Infernal for identification of 16S-containing reads, RDP classifier to classify reads, and MEGAN for visualization of results. [file 40168_2015_86_MOESM3_ESM.pdf]
